# On the Convulsions of New-born Children

**Published:** 1845-07

**Authors:** 


*On the Convulsions of New-born Children.*—The author only treats of the epileptic convulsions (epilepsia eclampsia neonatorum, Meissner,) and not of involuntary movements of the muscles of the face, eyes, or extremities. Meissner believes these convulsions to be rarely idiopathic, but most generally symptomatic, or reflex of diseases of the reproductive system. The observations collected in the Lying-in and Foundling Hospitals of Prague, led the author to the following results. Out of 2,500 infants only nine cases of well marked convulsions appeared, all of them caused by a derangement of the central nervous system. The disease might be regarded in almost all these cases as primary; but in two cases suppurative phlebitis umbilicalis seemed to be connected with the cause. In the other cases, hemorrhage was found to have occurred within the skull, or the cavity of the spinal marrow, on the *dura mater*, rarely between the membranes, and never in the substance of the brain. As regards the *causes* of the hemorrhage, nothing could be ascertained; neither difficult nor artificial labour, neither artificial nor natural birth, seemed to exert any influence. In all convulsions produced by affection of the brain, the temperature of the head was increased, the facial muscles distorted (as also in hydrencephalus,) and in two cases there was an erection of the penis; in convulsions, however, proceeding from affection of the spinal marrow, opisthotonos appeared, with contractions of the extremities; and increased temperature of the head was a later symptom, so that the seat of the hemorrhage may be presumed from these symptoms. The pulse could not be counted in any instance; the urine and fæces were rarely evacuated. The first attack never appeared before the second day after the birth, and one case only, on the 12 day, when the dissection distinctly showed that the extravasation of blood must have taken place several days before. [This shows that it is not every extravasation of blood that produces convulsions, but that it only favours convulsive tendency.] The paroxysms were repeated in some cases every three minutes, in others every hour. In the intervals, the children appeared in a state of stupor. The author did not see any child recover from real convulsions. The course of the disease was always very acute, varying from an hour to one or two days, when it invariably terminated fatally. A powerful antiphlogistic treatment seemed alone to have any beneficial influence—*Ibid, from Ibid.*

